# Quantitative Glandular Tissue Component Assessment on Breast Ultrasound Using an Anatomically Constrained Deep Learning Framework

**DOI:** 10.3390/diagnostics16203363

**Published:** 2026-10-15

**Authors:** Jun Jiang, Xin Zhou, Yi-Fan Wu, Xin-Liang Xu, Wen-Ying Li, Yue-Yang Guan, Yu Wang, Yi Dong

**Affiliations:** 1 Department of Ultrasound, Xinhua Hospital Affiliated to Shanghai Jiao Tong University School of Medicine, 1665 Kongjiang Road, Shanghai 200092, China; 2 Shanghai Yishu Huazhang Data Technology Co., Ltd., Shanghai 200092, China

**Keywords:** breast ultrasound, glandular tissue component, quantitative imaging, deep learning, artificial intelligence

## Abstract

**Background/Objectives:** Glandular tissue component (GTC) has been incorporated into BI-RADS v2025 as part of breast tissue composition assessment, but visual grading remains subjective and does not provide continuous quantification. We developed and evaluated an ROI-guided, anatomically constrained deep learning framework for quantitative GTC assessment on breast ultrasound. Reader performance was compared between unaided and AI-assisted sessions. **Methods:** This retrospective single-center study included 421 ultrasound images from 114 women for model development and 150 women for clinical evaluation. The framework segmented fibroglandular tissue (FGT) and glandular tissue to calculate continuous GTC percentage and assign the corresponding image-derived P1–P4 category. Four model configurations were compared during model development using patient-level five-fold cross-validation. Standalone AI performance was evaluated against expert consensus. Two senior and two junior radiologists assessed the clinical cohort without and with AI assistance, separated by a 4-week washout period. **Results:** During model development, the anatomical constraint markedly improved anatomical consistency while quantitative error and segmentation performance remained comparable. In the clinical cohort, continuous GTC percentage showed an MAE of 3.88 percentage points and strong correlation with the reference percentage (Spearman ρ = 0.957, *p* < 0.001). Exact P1–P4 agreement was 78.0%, adjacent agreement was 99.3%, and weighted κ was 0.888. However, 26 of 36 reference P4 cases (72.2%) were underestimated. Exact agreement was significantly higher for all four readers in the second, AI-assisted session than in the first, unaided session (all *p* < 0.001). **Conclusions:** The framework enabled anatomically consistent continuous GTC quantification and categorical assessment on individual breast ultrasound images.

## 1. Introduction

Breast tissue composition is an established imaging phenotype associated with breast cancer risk and imaging performance. Mammographic breast density is an independent risk factor for breast cancer and may reduce mammographic sensitivity through tissue masking [1,2], whereas breast MRI provides complementary measures of fibroglandular tissue (FGT) and background parenchymal enhancement [3,4,5]. The increasing use of automated and deep learning-based approaches across these modalities reflects a broader shift from subjective visual assessment toward more reproducible quantitative characterization of breast parenchyma [6,7,8,9,10,11].

Ultrasound-based glandular tissue component (GTC) has gained greater clinical relevance following its incorporation into BI-RADS v2025 as part of breast tissue composition assessment. BI-RADS v2025 characterizes sonographic breast tissue composition using tissue pattern and GTC, with GTC classified into four categories according to the proportion of glandular tissue within FGT [12,13]. Unlike mammographic density, in which glandular and fibrous tissues both contribute to radiographic density, ultrasound can distinguish glandular tissue from fibrous stroma within FGT based on their echogenic characteristics; therefore, women within the same mammographic density category may exhibit different sonographic GTC patterns [13,14]. Higher sonographic GTC has also been associated with less lobular involution and an increased risk of subsequent breast cancer in women with dense breasts [14,15,16]. These findings, together with the incorporation of GTC into BI-RADS v2025, highlight the need for standardized and reproducible GTC assessment.

Despite its increasing clinical relevance, GTC is still assessed mainly by visual estimation, with only moderate interobserver agreement reported for both four-category and dichotomous classifications [14,17]. Artificial intelligence (AI) has increasingly been used in breast ultrasound to reduce reader dependence and improve the consistency of image interpretation, with particular potential to assist less-experienced readers [18]. In GTC assessment specifically, both classification- and segmentation-based AI approaches have been investigated, together with AI-assisted reader assessment [19]. However, the reported evaluation focused on categorical grading rather than the accuracy of continuous gland-to-FGT measurements. Continuous quantification can complement categorical grading by preserving differences in tissue proportion within each category. Because this measurement depends on the relative areas of glandular tissue and FGT, explicitly accounting for their anatomical containment relationship may help limit implausible predictions and provide an anatomically consistent quantitative basis for grading.

Therefore, we developed an ROI-guided, anatomically constrained deep learning framework for quantitative GTC assessment on breast ultrasound. We evaluated its segmentation performance, anatomical consistency, and quantitative accuracy; assessed AI-derived GTC measurements in a clinical cohort; and compared P1–P4 classification performance between unaided and AI-assisted sessions among radiologists with different levels of experience.

## 2. Materials and Methods

### 2.1. Study Design and Population

This retrospective single-center study was approved by the institutional review board (XHEC-C-2026-073-1, approval date: 23 March 2026), which waived the requirement for written informed consent from patients because of the retrospective study design. Separate informed consent from the radiologists was not required. The study comprised two components: model development with technical evaluation and clinical evaluation with a reader study. Cohort sizes were determined by the availability of eligible cases. No formal a priori sample-size or precision calculation was performed for the clinical evaluation cohort.

The model development cohort comprised 421 breast ultrasound images from 114 women and was used for model development, technical evaluation, and stepwise ablation analysis. Multiple eligible images per patient could be included, and no formal balancing across GTC categories was applied. The clinical evaluation cohort comprised 150 women selected to achieve an approximately balanced distribution across the four image-level reference GTC categories, which had been established from the consensus masks before final image selection. One selected background breast parenchymal image was included per patient. Images were selected by a breast radiologist based on adequate image quality, clear visualization of the FGT boundaries, location away from the areolar region, and absence of focal lesions. Image selection was not blinded to GTC category but was performed without access to the final model output. There was no patient overlap between the two cohorts.

Images were retrospectively obtained from routine breast ultrasound examinations performed between October 2025 and February 2026 using systems from multiple manufacturers. Equipment details and manufacturer distributions are provided by cohort in Table S1, and available acquisition parameters are summarized in Table S2. Exclusion criteria were pregnancy or lactation, previous breast surgery, injection of foreign material into the breast, breast implants, and inadequate image quality. Because a complete screening log of all potentially eligible examinations was not available, the exact size of the source population and complete participant flow could not be fully reconstructed from the retrospective records.

### 2.2. Reference Standard and GTC Classification

The reference standard was established by two senior radiologists, each with more than 10 years of experience in breast ultrasound. To facilitate annotation, preliminary fibroglandular tissue (FGT) and glandular tissue masks were generated using an nnU-Net-based annotation model trained on a separate set of 100 breast ultrasound images with manually delineated masks. There was no patient overlap with either study cohort. The annotation model was frozen before generating masks for the study images, with no further training or tuning. One radiologist reviewed each preliminary mask and manually corrected it where necessary. A second radiologist independently reviewed the corrected annotations, and any disagreements were resolved by joint review. The resulting consensus masks served as the reference standard. These masks were finalized before model training for the development cohort and before application of the final model to the clinical evaluation cohort. Continuous GTC percentage was defined as the proportion of glandular tissue area within the FGT region on each ultrasound image. For the present image-level analysis, the GTC percentage was classified as minimal (<25%), mild (25% to <50%), moderate (50% to <75%), or marked (≥75%) using the specified percentage thresholds [12,13]. These image-derived categories were coded as P1, P2, P3, and P4, respectively. For the secondary binary analysis, P1–P2 were grouped as low GTC and P3–P4 as high GTC [14].

### 2.3. AI Framework and Image Preprocessing

**Figure 1:**
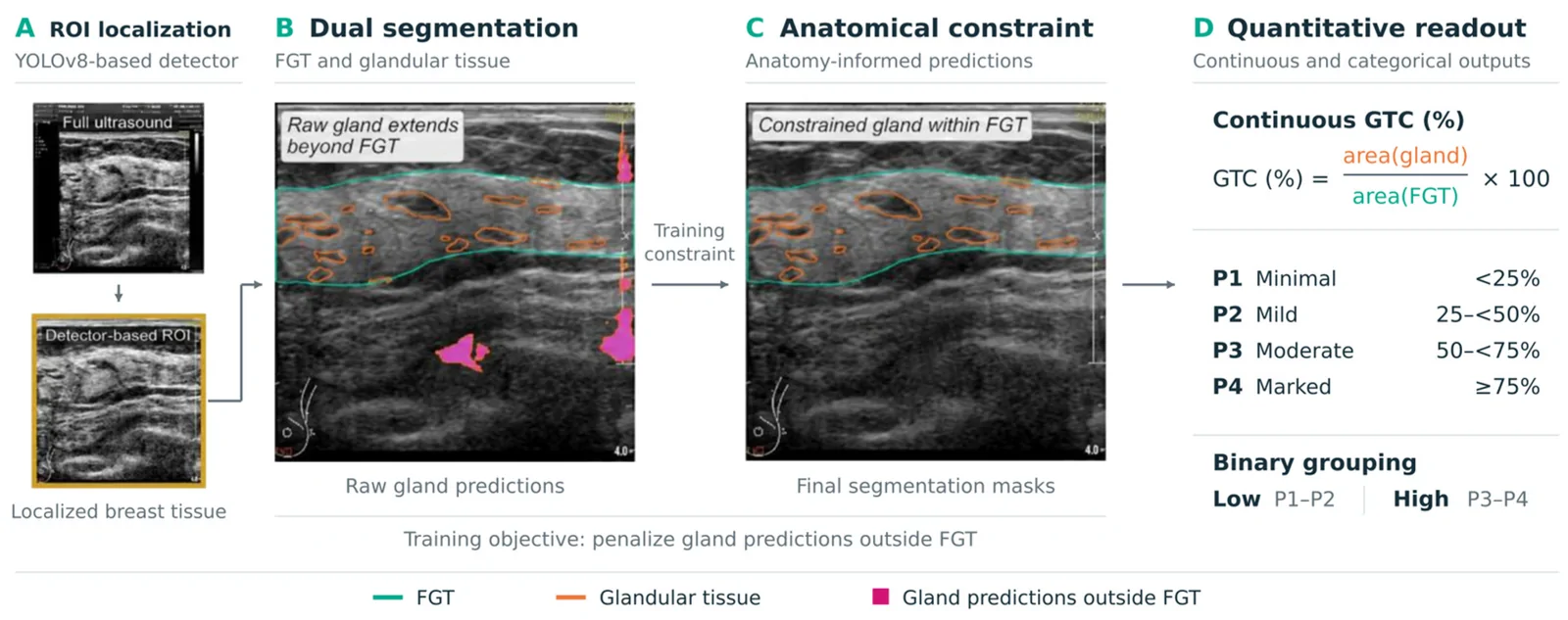
Workflow of the ROI-guided, anatomically constrained framework for quantitative GTC assessment on breast ultrasound. (**A**) A YOLOv8-based detector localizes the breast tissue region from the original ultrasound image. (**B**) Within the detected ROI, fibroglandular tissue (FGT) and glandular tissue are segmented; unconstrained raw gland predictions may extend beyond the predicted FGT region. (**C**) The anatomical consistency constraint penalizes gland probabilities exceeding the corresponding FGT probabilities during training, thereby promoting greater anatomical consistency of gland predictions. (**D**) The final segmentation masks are used to calculate the continuous glandular tissue component (GTC) percentage and assign the corresponding P1–P4 category and binary low/high classification. Teal denotes FGT, orange denotes glandular tissue, and magenta denotes gland predictions outside the FGT region.

We developed an ROI-guided, anatomically constrained deep learning framework for quantitative GTC assessment on breast ultrasound images (Figure 1). A YOLOv8-based detector first localized the breast tissue region, after which the detected region of interest was cropped and resized to 640 × 640 pixels. The same ROI localization and preprocessing pipeline was applied to all compared models. Details of ROI-detector development, training data, and annotations are provided in the Supplementary Methods.

Within the ROI, a cascade-shared segmentation network generated FGT and glandular tissue masks. Because glandular tissue is anatomically contained within the FGT region, an anatomical consistency constraint was incorporated during training to penalize gland predictions extending beyond the predicted FGT region. The final masks were used to calculate continuous GTC percentage and assign the corresponding P1–P4 category and low/high classification.

### 2.4. Model Development and Comparison

Model development was performed using patient-level five-fold cross-validation, with four folds used for training and the remaining fold used for evaluation in each iteration. All images from the same patient were assigned to the same fold.

All configurations used identical patient-level folds, precomputed ROI coordinates, and preprocessing procedures. Each fold was trained for 100 epochs (50 epochs for the DeepLabV3 baseline) without early stopping. The held-out fold was evaluated every five epochs, and the checkpoint with the highest gland Dice was retained; no separate inner validation subset was used for checkpoint selection. Because the evaluation fold contributed to checkpoint selection and the loss weights had been fixed during preliminary development using images from the development cohort, the original cross-validation is interpreted as a development-stage model comparison rather than an unbiased held-out estimate of performance. A sensitivity analysis using a patient-level inner validation subset for checkpoint selection was therefore performed (Supplementary Methods and Table S3). Detailed training and implementation settings are also provided in the Supplementary Methods.

Four configurations were compared. The Anchor model used the cascade-shared architecture with a ratio-loss weight of 0.05 and no anatomical consistency constraint. The +Constraint model retained the same architecture and ratio-loss weight and added an anatomical consistency loss with a weight of 0.05. The +Constraint + Ratio10 model retained the constraint and increased the ratio-loss weight to 0.10 to emphasize quantitative GTC estimation. A DeepLabV3 model with a ResNet50 backbone (torchvision deeplabv3_resnet50, torchvision 0.22, PyTorch 2.7), initialized with the COCO_WITH_VOC_LABELS_V1 pretrained weights, served as a conventional segmentation baseline without anatomical consistency constraints or ratio-oriented optimization [20]. The original 21-class output layer was replaced by two 1 × 1 convolutional heads for FGT and gland segmentation sharing the atrous spatial pyramid pooling features, with no auxiliary classifier. The anatomical-consistency and ratio-loss weights were fixed during preliminary development before the reported cross-validation; additional loss and training details are provided in the Supplementary Methods.

The three cascade-shared configurations formed a stepwise ablation analysis to assess the contributions of anatomical consistency modelling and stronger ratio-oriented optimization. The configuration used for clinical evaluation was selected during the original development procedure based on quantitative GTC error, anatomical consistency, and segmentation performance.

After selection of the final configuration, the model was retrained using the entire development cohort of 421 images, with the training duration determined from the cross-validation results as described in the Supplementary Methods. The model was frozen before first access to the clinical evaluation cohort and applied without further modification.

### 2.5. Technical Evaluation Metrics

Segmentation performance was assessed using Dice similarity coefficients for FGT and glandular tissue. Gland Dice was calculated only for images with non-empty reference gland masks; no reference FGT or gland mask was empty, and all technical evaluation metrics were therefore calculated on all 421 development images.

Anatomical consistency was assessed using the gland-outside-FGT ratio, calculated before final containment as |G_raw_ ∩ ¬F_pred_|/|G_raw_|, where G_raw_ and F_pred_ denote the thresholded raw gland and predicted FGT masks, respectively; the ratio was set to 0 when G_raw_ was empty. The final gland mask was obtained as G_final_ = G_raw_ ∩ F_pred_, so final containment was imposed by construction and was distinct from the reported gland-outside-FGT ratio. Quantitative performance was assessed using GTC area-ratio mean absolute error (MAE), defined as the absolute difference between predicted and reference gland-to-FGT area ratios. Metrics were calculated for each evaluation fold and summarized across the five folds. All 421 development images received successful ROI localization and a non-empty FGT prediction in every configuration; there were no missing predictions, no images were excluded after processing, and no fallback or manual correction was required during model inference.

### 2.6. Clinical Evaluation and Reader Study

Standalone AI and reader performance were evaluated in the clinical evaluation cohort. Four radiologists were selected based on availability: two senior and two junior radiologists with 8, 6, 2, and 1 years of breast ultrasound experience, respectively.

In the first session, readers independently reviewed the images without AI assistance and assigned P1–P4 categories. After a 4-week washout period, the same images were presented in a newly randomized order and reassessed with AI assistance. The interface displayed the AI-derived continuous GTC percentage, P1–P4 category, and low/high classification, and readers could accept or modify the AI-suggested category.

Readers were blinded to the reference standard, other readers’ assessments, and their own previous interpretations. P1–P4 categories were used for the primary reader-performance analysis, while low/high classifications were derived from the corresponding categories.

### 2.7. Statistical Analysis

Technical cross-validation metrics are presented as mean ± standard deviation across the five evaluation folds. In the clinical evaluation cohort, quantitative error in AI-derived GTC percentage was assessed using MAE, and its association with the reference GTC percentage was evaluated using Spearman rank correlation. Mean bias was calculated as AI-derived minus reference GTC percentage for the overall cohort and reference P4 cases.

Four-category performance was assessed using exact agreement, adjacent agreement, and quadratic weighted Cohen’s κ. Adjacent agreement included exact agreement and one-category discrepancies. For the prespecified secondary low/high analysis, accuracy, sensitivity, and specificity were calculated, with high GTC considered the positive class. Standalone AI performance estimates are presented with 95% confidence intervals. For MAE, mean bias, Spearman rank correlation, and quadratic weighted κ, confidence intervals were obtained using patient-level nonparametric percentile bootstrap resampling; exact binomial confidence intervals were used for proportion-based metrics.

Reader performance before and after AI assistance was evaluated using exact agreement and quadratic weighted Cohen’s κ. Paired changes in exact-agreement status were assessed using the McNemar test. Inter-reader agreement was assessed using Fleiss’ κ for both P1–P4 and low/high classifications. Reader exact-agreement proportions are presented with 95% confidence intervals. Comparisons between senior and junior readers were descriptive. All tests were two-sided, with *p* < 0.05 indicating statistical significance. Statistical analyses were performed using SPSS software (version 25.0; IBM Corp., Armonk, NY, USA) and R software (version 4.5.1; R Foundation for Statistical Computing, Vienna, Austria).

## 3. Results

### 3.1. Technical Performance of the Model Configurations

The model development cohort included 421 breast ultrasound images from 114 women. The original development-stage comparison across the four model configurations is summarized in Table 1. FGT Dice remained comparable across the four configurations, and gland Dice was broadly similar among the three cascade-shared models, whereas DeepLabV3-ResNet50 showed lower gland segmentation performance.

**Figure 2:**
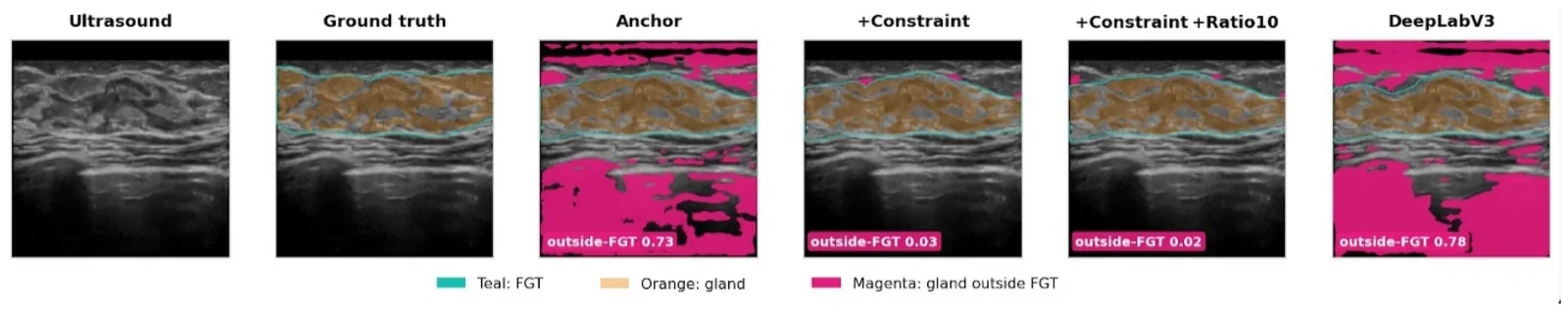
Representative comparison of pre-cascade gland-outside-FGT predictions across model configurations. The original ultrasound image and reference annotation are shown alongside predictions from the Anchor, +Constraint, +Constraint + Ratio10, and DeepLabV3-ResNet50 models. The displayed outside-FGT values reflect the extent of pre-cascade gland predictions extending beyond the predicted fibroglandular tissue region, with lower values indicating better anatomical consistency.

The principal effect of anatomical consistency modelling was observed in the gland-outside-FGT ratio, which decreased from 0.7043 ± 0.0299 in the Anchor model to 0.1816 ± 0.0891 after introduction of the anatomical constraint and to 0.1579 ± 0.0559 in the +Constraint + Ratio10 model. The representative example likewise showed fewer pre-cascade gland predictions extending beyond the predicted FGT region in the constrained models than in the Anchor and DeepLabV3-ResNet50 models (Figure 2).

In the original development-stage comparison, the GTC area-ratio MAE of +Constraint + Ratio10 was similar to that of the Anchor model (0.0557 ± 0.0076 vs. 0.0568 ± 0.0063). The inner-validation sensitivity analysis further showed that the gland-outside-FGT ratio remained markedly lower for +Constraint + Ratio10 than for the Anchor model (0.2299 ± 0.0737 vs. 0.7038 ± 0.0454), while their GTC area-ratio MAEs were comparable (0.0456 ± 0.0046 vs. 0.0441 ± 0.0033; Supplementary Table S3). Taken together, both analyses showed that the anatomical constraint reduced anatomically inconsistent raw gland predictions, while the GTC area-ratio MAE of +Constraint + Ratio10 remained comparable to that of the Anchor model.

### 3.2. Standalone AI Performance in the Clinical Evaluation Cohort

**Figure 3:**
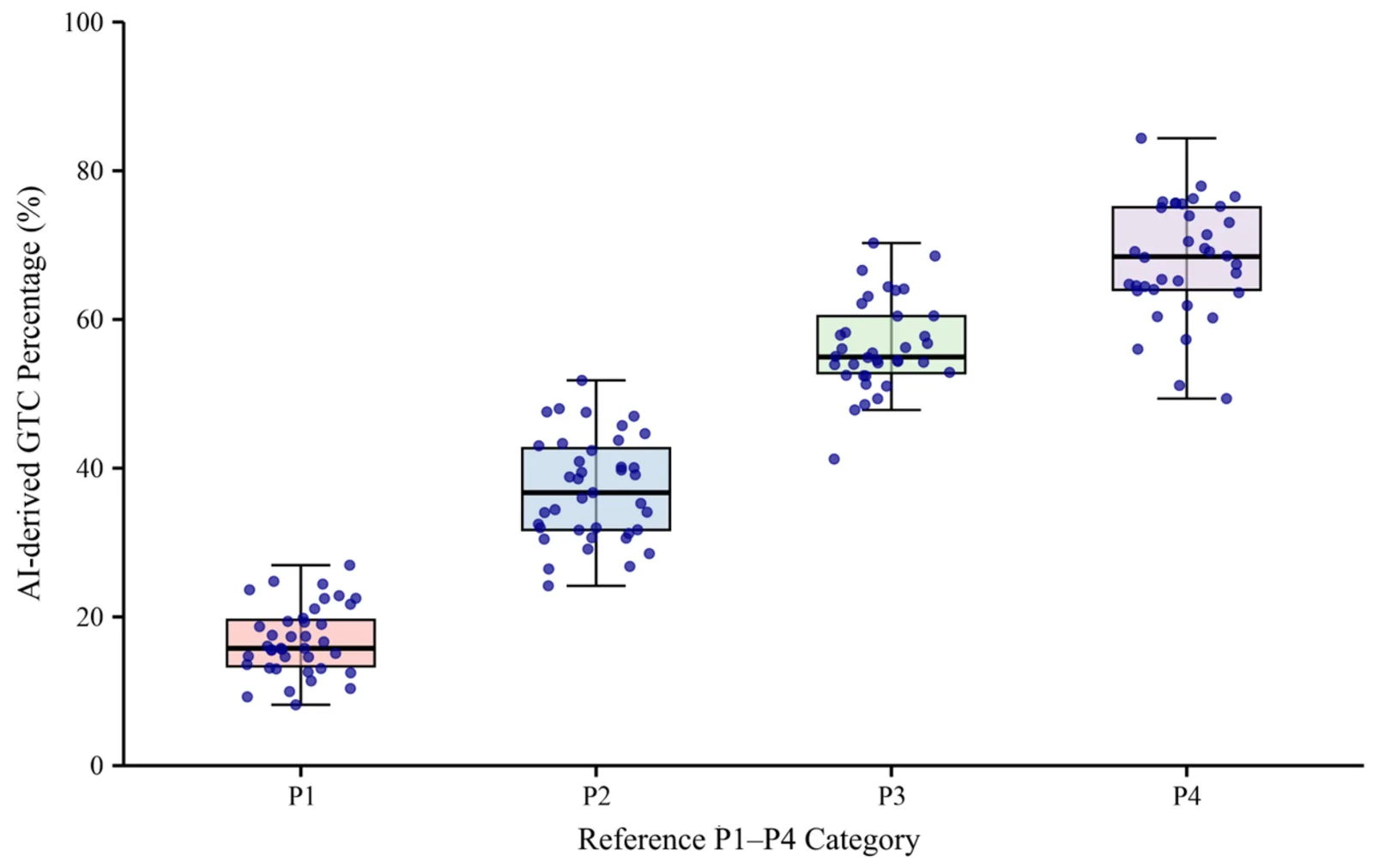
Distribution of AI-derived continuous GTC percentages across reference-defined P1–P4 categories in the clinical evaluation cohort. Individual points represent patients, and boxplots indicate the median and interquartile range.

The clinical evaluation cohort included 150 women, with reference GTC categories comprising 39 P1, 39 P2, 36 P3, and 36 P4 cases. The standalone AI performance for continuous GTC percentage estimation and P1–P4 classification is summarized in Table 2. The mean absolute error for continuous GTC percentage was 3.88 percentage points, and the AI-derived and reference GTC percentages were strongly correlated (Spearman ρ = 0.957, *p* < 0.001). The AI-derived continuous GTC percentage increased progressively across the reference-defined P1–P4 categories (Figure 3). The overall mean bias was −3.38 percentage points (95% CI, −4.43 to −2.40), indicating slight underestimation on average.

For P1–P4 classification, the AI model achieved exact agreement in 117 of 150 cases (78.0%), with a quadratic weighted κ of 0.888. Adjacent agreement was observed in 149 of 150 cases (99.3%), indicating that nearly all discrepancies were confined to neighboring categories. In the secondary binary analysis, the AI model achieved an accuracy of 96.0%, a sensitivity of 93.1%, and a specificity of 98.7%. Among the 36 reference P4 cases, 10 (27.8%) were correctly classified and 26 (72.2%) were underestimated: 25 as P3 and one as P2 (Table 3). The mean bias in this subgroup was −10.32 percentage points (95% CI, −13.17 to −7.62).

### 3.3. Reader Performance and Inter-Reader Agreement

Exact agreement was significantly higher for all four readers in the second, AI-assisted session than in the first, unaided session (all McNemar *p* < 0.001), and quadratic weighted κ was also higher for each reader (Table 4). The average increase in exact agreement was descriptively greater among the two junior readers than among the two senior readers (18.0 vs. 12.3 percentage points). Average quadratic weighted κ increased from 0.713 to 0.853 among junior readers and from 0.897 to 0.947 among senior readers.

Inter-reader agreement was also higher in the second, AI-assisted session. For P1–P4 classification, Fleiss’ κ increased from 0.355 in the unaided session to 0.592 in the AI-assisted session. For binary low/high classification, Fleiss’ κ increased from 0.599 to 0.822.

## 4. Discussion

In this study, we developed and evaluated an ROI-guided, anatomically constrained deep learning framework for quantitative GTC assessment on breast ultrasound. The anatomical constraint markedly reduced raw gland predictions extending beyond the FGT region without materially compromising segmentation performance. In the clinical evaluation cohort, AI-derived continuous GTC percentages showed low quantitative error and were strongly correlated with the reference measurements, while the corresponding P1–P4 categories showed strong weighted agreement, with nearly all discrepancies confined to adjacent categories. Exact agreement was significantly higher for all four readers in the AI-assisted session, with descriptively larger gains among junior readers. Collectively, these findings support the feasibility of integrating anatomically constrained segmentation, continuous measurement, and categorical assessment within a single GTC assessment framework.

Unlike mammographic density, which reflects the combined amount of glandular and fibrous tissue relative to fat, sonographic GTC characterizes the relative glandular component within FGT [14]. Previous studies have associated higher sonographic GTC with less extensive lobular involution and an increased subsequent risk of breast cancer among women with mammographically dense breasts [14,15]. Its incorporation into BI-RADS v2025 further increases the need for reproducible and standardized assessment in clinical practice [12,13]. However, the present study addressed GTC quantification and classification on individual ultrasound images; it did not evaluate whole-breast GTC, breast cancer risk, or patient outcomes; instead, it addressed the methodological question of whether GTC can be quantitatively measured and consistently classified on individual breast ultrasound images.

A major limitation of the conventional GTC assessment is its dependence on visual estimation. Previous four-category assessment of GTC on breast ultrasound demonstrated moderate interobserver agreement, highlighting the variability inherent in qualitative tissue-composition grading [17]. Deep learning has also been used to infer mammographic density from clinical breast ultrasound images, suggesting that ultrasound contains quantifiable information related to breast tissue composition [21]. More recently, AI-assisted approaches have been explored for sonographic GTC classification, with improved P1–P4 grading performance reported among breast and non-breast radiologists [19]. However, the reported evaluation focused on categorical performance rather than the accuracy of continuous gland-to-FGT measurements. Our framework combines continuous gland-to-FGT quantification with an explicit anatomical constraint that penalizes gland predictions outside FGT. The continuous area ratio is then used to derive the P1–P4 category, providing a measurable tissue proportion underlying each categorical assignment.

Because GTC percentage is derived from the relative areas of two predicted compartments, it depends on the relationship between the gland and FGT predictions. In all configurations, the final gland mask was contained within the predicted FGT by construction, so raw gland predictions outside the predicted FGT did not enter the GTC calculation directly. In the ablation analysis, the anatomical constraint substantially reduced the gland-outside-FGT ratio before containment while preserving broadly comparable Dice coefficients, indicating improved anatomical consistency of the raw gland output rather than improved containment of the final gland mask. Across the original and inner-validation analyses, this reduction was preserved, while the GTC area-ratio MAE of +Constraint + Ratio10 remained comparable to that of the Anchor model. These findings support considering anatomical consistency alongside Dice during model development.

The clinical evaluation characterized model performance at complementary continuous and categorical levels. The strong correlation between AI-derived and reference GTC percentages, together with the progressive increase in AI-derived values from P1 to P4, indicated that the continuous output preserved the ordered structure of the four-category GTC classification. Continuous measurement also retained within-category information that was not represented by the four categories alone. However, conversion of continuous values into four categories using fixed thresholds may result in adjacent-category discrepancies when estimates are close to category boundaries.

In the present cohort, 26 of 36 reference P4 cases were underestimated, with a mean bias of −10.32 percentage points across all reference P4 cases. Of these 26 cases, 25 were classified as P3 and one as P2. Thus, high weighted κ and adjacent agreement coexisted with limited exact classification of P4. Because P3 and P4 both belong to the high-GTC group, 25 of these errors did not alter the binary classification, consistent with the overall low/high accuracy of 96.0%. These findings highlight good performance for the broader binary distinction, although precise classification at the upper end of the GTC range remains a limitation.

In the AI-assisted session, all four readers showed higher exact agreement and weighted κ, with higher inter-reader agreement. The descriptively larger gains observed among junior radiologists were consistent with previous AI-assisted reader studies showing greater benefits among less-experienced readers [22,23,24,25]. AI-assisted breast ultrasound has also been reported to reduce inter-reader variability and improve the consistency of interpretation [23], while recent evidence highlights its potential to provide more standardized and reproducible decision support [18]. Because readers could accept or modify the AI-suggested category, the assisted assessments represented human–AI decision making rather than fully automated classification, consistent with current European practice recommendations that AI should complement rather than replace radiologist interpretation [26].

This study has several limitations. First, it was retrospective and conducted at a single center. The clinical evaluation cohort was selected to provide an approximately balanced GTC distribution and therefore does not reflect the category distribution of a consecutive clinical population. Although images were acquired using ultrasound systems from multiple manufacturers, external multicenter validation is required to assess generalizability across institutions, patient populations, acquisition protocols, and operators. Second, ROI-detector development used an image-based rather than patient-level split and partially overlapped with the segmentation development cohort, limiting the independence of the upstream preprocessing in the technical cross-validation. Third, model performance was less accurate at the upper end of the GTC range, with a tendency to underestimate reference-defined P4 cases. Further model refinement and focused validation in high-GTC cases are warranted. Fourth, the offline reader study included only four readers and used a fixed session order. Despite the four-week washout and re-randomization of case order, the contribution of AI assistance could not be separated from order and repeat-reading effects. Randomized or counterbalanced studies with more readers are needed to establish its contribution and clinical value.

In conclusion, the ROI-guided, anatomically constrained framework enabled continuous and categorical GTC assessment on individual breast ultrasound images while preserving the anatomical relationship between glandular tissue and FGT. The model showed high overall weighted agreement with the reference assessments, but underestimated most reference P4 cases. Readers showed better performance and higher inter-reader agreement in the AI-assisted session. External validation and prospective clinical evaluation are needed before broader clinical application.

## Figures and Tables

**Table 1:** Development-stage comparison of the four model configurations using five-fold cross-validation for quantitative GTC assessment on breast ultrasound.

Method	FGT Dice	Gland Dice	Gland-Outside-FGT Ratio	GTC Area-Ratio MAE
Anchor	0.9402 ± 0.0186	0.7738 ± 0.0281	0.7043 ± 0.0299	0.0568 ± 0.0063
+Constraint	0.9422 ± 0.0121	0.7791 ± 0.0228	0.1816 ± 0.0891	0.0638 ± 0.0235
+Constraint + Ratio10	0.9393 ± 0.0198	0.7750 ± 0.0246	0.1579 ± 0.0559	0.0557 ± 0.0076
DeepLabV3-ResNet50	0.9418 ± 0.0179	0.7558 ± 0.0208	0.7448 ± 0.0396	0.0819 ± 0.0164

FGT, fibroglandular tissue; GTC, glandular tissue component; MAE, mean absolute error.

**Table 2:** Standalone performance of the AI model in the clinical evaluation cohort.

Performance Metric	Result	95% CI
GTC percentage MAE	3.88 percentage points	2.94–4.89
Mean bias in GTC percentage	−3.38 percentage points	−4.43 to −2.40
Spearman correlation	0.957, *p* < 0.001	0.922–0.978
P1–P4 exact agreement	117/150, 78.0%	70.5–84.3%
P1–P4 adjacent agreement	149/150, 99.3%	96.3–100.0%
Quadratic weighted κ for P1–P4 classification	0.888	0.845–0.923
Low/high accuracy	96.0%	91.5–98.5%
Low/high sensitivity	93.1%	84.5–97.7%
Low/high specificity	98.7%	93.1–100.0%

AI, artificial intelligence; GTC, glandular tissue component; MAE, mean absolute error; CI, confidence interval. Bias = AI-derived − reference GTC percentage.

**Table 3:** Confusion matrix of AI-assigned and reference-defined P1–P4 categories.

	AI P1	AI P2	AI P3	AI P4	Total
Reference P1	38	1	0	0	39
Reference P2	1	37	1	0	39
Reference P3	0	4	32	0	36
Reference P4	0	1	25	10	36
Total	39	43	58	10	150

AI, artificial intelligence.

**Table 4:** Reader performance before and after AI assistance.

Reader	Unaided Exact Agreement, *n*/N, % (95% CI)	AI-Assisted Exact Agreement, *n*/N, % (95% CI)	Unaided Weighted κ	AI-Assisted Weighted κ	Incorrect → Correct, *n*	Correct → Incorrect, *n*	McNemar *p*
Reader 1 (Senior)	113/150, 75.3% (67.6–82.0)	133/150, 88.7% (82.5–93.3)	0.907	0.954	22	2	<0.001
Reader 2 (Senior)	114/150, 76.0% (68.4–82.6)	131/150, 87.3% (80.9–92.2)	0.887	0.940	20	3	<0.001
Reader 3 (Junior)	88/150, 58.7% (50.3–66.6)	114/150, 76.0% (68.4–82.6)	0.804	0.892	33	7	<0.001
Reader 4 (Junior)	68/150, 45.3% (37.2–53.7)	96/150, 64.0% (55.8–71.7)	0.621	0.814	37	9	<0.001

AI, artificial intelligence; CI, confidence interval. Weighted κ indicates quadratic weighted kappa. Arrows indicate changes from unaided to AI-assisted assessment. McNemar *p* values compare unaided and AI-assisted exact agreement.

## Data Availability

The datasets generated and/or analyzed during the current study are available from the corresponding author upon reasonable request.

## Supplementary Materials

The following supporting information can be downloaded at: https://www.mdpi.com/article/10.3390/diagnostics16203363/s1, Supplementary Methods; Table S1: Ultrasound equipment and manufacturer distribution by cohort; Table S2: Available ultrasound acquisition parameters by cohort; Table S3: Sensitivity analysis using inner-validation checkpoint selection.

## Abbreviations

The following abbreviations are used in this manuscript:

AI	Artificial intelligence
CI	Confidence interval
FGT	Fibroglandular tissue
GTC	Glandular tissue component
MAE	Mean absolute error
MRI	Magnetic resonance imaging
ROI	Region of interest
