# Supplementary material for: Quantitative Glandular Tissue Component Assessment on Breast Ultrasound Using an Anatomically Constrained Deep Learning Framework
Source: Diagnostics (Basel). 2026 Oct 15;16(20):3363. doi: 10.3390/diagnostics16203363 (PMC13702955; doi:10.3390/diagnostics16203363)
Supplement: Supplementary file 1 [file diagnostics-16-03363-s001.zip › diagnostics-4583826-supplementary.pdf]

Supplementary Materials

Supplementary Methods

1. Model training and checkpoint selection

Model development used patient-level five-fold cross-validation. For the cascade-shared configurations, each fold was trained for a fixed 100 epochs without early stopping; the DeepLabV3-ResNet50 baseline was trained for 50 epochs. During the original cross-validation, the held-out fold was evaluated every five epochs, and the checkpoint with the highest gland Dice was retained. No separate inner validation subset was used for checkpoint selection.

All models were trained using AdamW with a learning rate of 1×10^{-4}, weight decay of 1×10^{-4}, cosine-annealing learning-rate scheduling, a batch size of 4, and an input size of 640 × 640 pixels. A single random seed (42) was used. Data augmentation included random horizontal and vertical flipping, 90° rotation, affine transformation, elastic deformation, and grid distortion, followed by ImageNet normalization. At inference, both probability maps were thresholded at 0.5, the final gland mask was constrained by the predicted FGT mask, and no additional post-processing was applied. These settings were kept consistent across the compared configurations except for the predefined architectural and loss-function differences.

The anatomical-consistency loss weight (0.05) and the ratio-loss weights (0.05 and 0.10) were fixed during preliminary development using development-cohort images before the reported cross-validation. They were not adjusted during the reported cross-validation or using information from the clinical evaluation cohort; however, this preliminary hyperparameter selection was not nested within the outer cross-validation.

The DeepLabV3-ResNet50 baseline used torchvision deeplabv3_resnet50 initialized with COCO_WITH_VOC_LABELS_V1 pretrained weights. The original classification head was replaced by two 1 × 1 convolutional heads for FGT and gland segmentation, sharing the ASPP features, with no auxiliary classifier.

2. ROI detector development

A YOLOv8-nano detector with COCO-pretrained initialization was used for ROI localization. The detector was developed using 72 grayscale breast ultrasound images annotated with a single rectangular scan-area bounding box. The detector-development split was image-based rather than patient-level. Twenty-nine detector-

development images also occurred in the segmentation development cohort; however, the detector annotations contained only the scan-area bounding box and did not include FGT or glandular-tissue masks. No patient used for ROI-detector development was included in the clinical evaluation cohort.

The detector was frozen before segmentation-model evaluation, and the same precomputed ROI coordinates were used for all compared model configurations. All 421 development images received successful ROI localization, and no fallback localization procedure was required. Validation performance of the detector was mAP50 = 0.995. Given the image-based development split and partial overlap with the segmentation development cohort, the upstream ROI preprocessing used in the technical cross-validation was not fully independent of the evaluation folds.

3. Sensitivity analysis of checkpoint selection

Because the original cross-validation used the held-out evaluation fold for checkpoint selection, an additional sensitivity analysis was performed in which checkpoint selection was moved to a patient-level inner validation subset drawn from the training portion of each outer fold.

All four model configurations were re-evaluated. Apart from the location of checkpoint selection, the analysis retained the same frozen ROI detector and precomputed ROI coordinates, model architectures, loss weights, training budget, random seed, checkpoint-selection criterion, and outer-fold evaluation procedure. The outer held-out fold was not used for checkpoint selection in this sensitivity analysis.

The marked reduction in gland-outside-FGT ratio associated with the anatomical constraint was preserved when checkpoint selection was performed without access to the outer evaluation fold, while GTC area-ratio MAE remained comparable between +Constraint + Ratio10 and the Anchor model. Detailed results are shown in Table S3.

4. Final-model training and lock

After selection of the +Constraint + Ratio10 configuration, the final model was retrained using the entire development cohort of 421 images with the same model architecture, loss weights, preprocessing, and optimization settings.

Because no held-out validation data remained during full-cohort retraining, the training duration was fixed before retraining as the median of the five fold-specific best epochs identified during cross-validation. The resulting model weights were then frozen.

The clinical evaluation cohort was first accessed only after the final model had been

frozen. There was no patient overlap between the development and clinical evaluation cohorts, and no patient used for ROI-detector development was included in the clinical evaluation cohort. The same locked model was subsequently used to generate the standalone AI results and the AI outputs provided in the reader study.

5. Network architecture, loss functions, and mask generation

Architecture. The cascade-shared segmentation network used a shared EfficientNet-B3 encoder with ImageNet initialization and two parameter-independent U-Net decoders of identical structure for FGT and glandular-tissue segmentation. Both decoders received the same multi-scale encoder features through skip connections, but no features were exchanged between them. Each decoder produced a single-channel logit map, z_F for FGT and z_G for glandular tissue, with corresponding FGT and gland probability maps denoted by p_F and p_G, respectively:

$$p_F = \sigma(z_F), \qquad p_G = \sigma(z_G).$$

In this implementation, “shared” refers to the common encoder, whereas “cascade” refers to the hierarchical coupling of gland prediction to FGT through FGT-restricted gland supervision, coupled anatomical-consistency and ratio losses, and final containment at inference rather than feature transfer between the two decoders.

Loss functions. Let F and G denote the reference FGT and gland masks, respectively, and Ω the pixels within the 640 × 640 ROI. For a mini-batch of $B = 4$ images and epoch index $e = 0, 1, \ldots$, the warm-up factor was

$$w(e) = \min\left[1, \frac{e+1}{5}\right].$$

The segmentation loss was

$$L_{\text{seg}} = 0.2w(e)[L_{\text{Lov}}(z_F, F; \Omega) + L_{\text{Lov}}(z_G, G; F)] + 0.1L_{\text{FP},F} + 0.1w(e)L_{\text{FP},G},$$

where L_{Lov} denotes the Lovász hinge loss, with the gland term evaluated within the reference FGT region. The Lovász loss was computed per image and averaged over the batch. The false-positive penalties were

$$L_{\text{FP},F} = \frac{1}{B|\Omega|}\sum_b \sum_x p_F(x)[1 - F(x)],$$

and

$$L_{\mathrm{FP},G} = \frac{\sum_b \sum_x p_G(x)[1 - G(x)]F(x)}{\sum_b \sum_x F(x)}.$$

The anatomical-consistency loss was

$$L_{\mathrm{con}} = \frac{1}{B|\Omega|} \sum_b \sum_x \max\{0, p_G(x) - \mathrm{sg}[p_F(x)]\},$$

where $\mathrm{sg}[\cdot]$ denotes stop-gradient. The gradient of this term propagated through the gland branch and shared encoder but not through the FGT output.

The soft predicted GTC used for the ratio loss was

$$\mathrm{GTC}_{\mathrm{soft}} = \frac{\sum_x p_G(x) p_F(x)}{\sum_x p_F(x) + 10^{-6}},$$

with reference GTC

$$\mathrm{GTC}_{\mathrm{ref}} = \frac{|G \cap F|}{|F|},$$

and the ratio loss

$$L_{\mathrm{ratio}} = \frac{1}{B} \sum_b |\mathrm{GTC}_{\mathrm{soft}} - \mathrm{GTC}_{\mathrm{ref}}|.$$

The total loss was

$$L = L_{\mathrm{seg}} + \lambda_{\mathrm{con}} L_{\mathrm{con}} + w(e) \lambda_{\mathrm{ratio}} L_{\mathrm{ratio}},$$

with $(\lambda_{\mathrm{con}}, \lambda_{\mathrm{ratio}}) = (0, 0.05)$ for Anchor, $(0.05, 0.05)$ for +Constraint, and $(0.05, 0.10)$ for +Constraint + Ratio10.

Mask generation and evaluation. At inference, the FGT and raw gland masks were obtained as

$$F_{\mathrm{pred}} = [p_F > 0.5], \qquad G_{\mathrm{raw}} = [p_G > 0.5].$$

The final gland mask was defined as

$$G_{\mathrm{final}} = G_{\mathrm{raw}} \cap F_{\mathrm{pred}}.$$

This containment operation was applied to all model configurations. No morphological post-processing, connected-component filtering, or hole filling was applied. Predicted GTC was calculated as

$$\mathrm{GTC_{pred}} = \frac{|G_{\mathrm{final}}|}{|F_{\mathrm{pred}}|}.$$

Gland Dice and GTC area-ratio MAE in Table 1 and Supplementary Table S3 were calculated using G_{final}, whereas the gland-outside-FGT ratio was calculated before containment as

$$\frac{|G_{\mathrm{raw}} \cap \neg F_{\mathrm{pred}}|}{|G_{\mathrm{raw}}|},$$

with the ratio set to 0 when G_{raw} was empty. Thus, the gland-outside-FGT ratio reflects anatomical consistency of the raw gland prediction rather than containment of the final mask.

Supplementary Table S1. Ultrasound equipment and manufacturer distribution by cohort.

Manufacturer	Scanner model	Transducer model	Development cohort, images, n (%)	Clinical evaluation cohort, images, n (%)
Siemens Healthineers	ACUSON Sequoia Silver M	18L6	173 (41.1)	38 (25.3)
SonoScape	S70i	12L-A	127 (30.2)	72 (48.0)
GE HealthCare	LOGIQ Fortis	L3-12	86 (20.4)	21 (14.0)
Philips	iU22	L12-5	19 (4.5)	17 (11.3)
Samsung Medison	RS80A	L3-12A	16 (3.8)	2 (1.3)
Total			421	150

Manufacturer locations: Siemens Medical Solutions USA, Inc., Issaquah, WA, USA; SonoScape Medical Corp., Shenzhen, China; GE Ultrasound Korea, Ltd., Seongnam-si, Republic of Korea; Philips Ultrasound, Inc., Bothell, WA, USA; Samsung Medison Co., Ltd., Seoul, Republic of Korea.

Supplementary Table S2. Available ultrasound acquisition parameters by cohort.

Scanner model	Cohort	Imaging depth (cm), range	Gain, range*	Dynamic range
ACUSON Sequoia Silver M	Development	2.5–4.5	0–14 dB	65–70
	Clinical evaluation	3.0–4.5	0–8 dB	65–70
SonoScape S70i	Development	2.5–5.0	46–63	190–200
	Clinical evaluation	3.0–5.0	45–67	190–200
GE LOGIQ Fortis	Development	3.3–4.5	54–60	63–69
	Clinical evaluation	2.8–5.0	53–81	63–69
Philips iU22	Development	4.0–5.0	57–82%	58–66
	Clinical evaluation	2.5–5.0	56–84%	51–66
Samsung RS80A	Development	3.0–4.0	56–63	55
	Clinical evaluation	3.5–4.0	56–62	55

* Gain values are reported according to the manufacturer-specific display scale and are not directly comparable across ultrasound systems. Siemens gain is displayed in dB, Philips gain as a percentage, and other systems use manufacturer-specific numerical scales.

Supplementary Table S3. Sensitivity analysis using inner-validation checkpoint selection.

Configuration	Metric	Original Table 1	Inner-validation analysis
Anchor	GTC area-ratio MAE	0.0568 ± 0.0063	0.0441 ± 0.0033
	Gland-outside-FGT ratio	0.7043 ± 0.0299	0.7038 ± 0.0454
	FGT Dice	0.9402 ± 0.0186	0.9587 ± 0.0041
	Gland Dice	0.7738 ± 0.0281	0.8016 ± 0.0248
+Constraint	GTC area-ratio MAE	0.0638 ± 0.0235	0.0511 ± 0.0085
	Gland-outside-FGT ratio	0.1816 ± 0.0891	0.2317 ± 0.0682
	FGT Dice	0.9422 ± 0.0121	0.9569 ± 0.0039
	Gland Dice	0.7791 ± 0.0228	0.7996 ± 0.0259
+Constraint + Ratio10	GTC area-ratio MAE	0.0557 ± 0.0076	0.0456 ± 0.0046
	Gland-outside-FGT ratio	0.1579 ± 0.0559	0.2299 ± 0.0737
	FGT Dice	0.9393 ± 0.0198	0.9584 ± 0.0019
	Gland Dice	0.7750 ± 0.0246	0.8013 ± 0.0259
DeepLabV3-ResNet50	GTC area-ratio MAE	0.0819 ± 0.0164	0.0527 ± 0.0068
	Gland-outside-FGT ratio	0.7448 ± 0.0396	0.7693 ± 0.0261
	FGT Dice	0.9418 ± 0.0179	0.9579 ± 0.0032
	Gland Dice	0.7558 ± 0.0208	0.7899 ± 0.0224

Values are mean ± standard deviation across the five outer folds. In the sensitivity analysis, checkpoint selection was performed using a patient-level inner validation subset drawn from the training portion of each outer fold.
